# PPAR-****γ**** Impairment Alters Peroxisome Functionality in Primary Astrocyte Cell Cultures

**DOI:** 10.1155/2014/546453

**Published:** 2014-03-04

**Authors:** Lorenzo Di Cesare Mannelli, Matteo Zanardelli, Laura Micheli, Carla Ghelardini

**Affiliations:** Dipartimento di Neuroscienze, Psicologia, Area del Farmaco e Salute del Bambino—(Neurofarba)—Sezione di Farmacologia e Tossicologia, Università di Firenze, Viale Pieraccini 6, 50139 Florence, Italy

## Abstract

Peroxisomes provide glial cells with protective functions against the harmful effects of H_2_O_2_ on neurons and peroxisome impairment results in nervous lesions. Agonists of the **γ**-subtype of the Peroxisome-Proliferator-Activated-Receptors (PPAR) have been proposed as neuroprotective agents in neurodegenerative disorders. Nevertheless, the role of PPAR-**γ** alterations in pathophysiological mechanisms and the relevance of peroxisome functions in the PPAR-**γ** effects are not yet clear. In a primary cell culture of rat astrocytes, the irreversible PPAR-**γ** antagonist GW9662 concentration-dependently decreased the activity of catalase, the most important antioxidant defense enzyme in peroxisomes. Catalase functionality recovered in a few days and the PPAR-**γ** agonist rosiglitazone promoted reversal of enzymatic damage. The reversible antagonist G3335 reduced both the activity and expression of catalase in a rosiglitazone-prevented manner. G3335 reduced also the glutathione reductase expression, indicating that enzyme involved in glutathione regeneration was compromised. Neither the PPAR-**α** target gene Acyl-Coenzyme-A-oxidase-1 nor the mitochondrial detoxifying enzyme NADH:ubiquinone-oxidoreductase (NDFUS3) was altered by PPAR-**γ** inhibition. In conclusion, PPAR-**γ** inhibition induced impairment of catalase in astrocytes. A general decrease of the antioxidant defenses of the cell suggests that a PPAR-**γ** hypofunction could participate in neurodegenerative mechanisms through peroxisomal damage. This series of experiments could be a useful model for studying compounds able to restore peroxisome functionality.

## 1. Introduction

Hydrogen peroxide (H_2_O_2_) is ascribed to Reactive Oxygen Species (ROS), although it has no unpaired electrons. It can be formed by the dismutation reaction of O_2_^•−^ via the hydroperoxyl radical. Although H_2_O_2_ is not harmful, its conversion, through the Fenton reaction catalyzed by metal ions, generates the hydroxyl radical (^•^OH), probably the most highly reactive and toxic form of oxygen [1–3]. Catalase is a heme-containing peroxisomal enzyme that breaks down hydrogen peroxide to water and oxygen and is a main antioxidant defense [4, 5]. De Duve and Baudhuin [6] first described a respiratory pathway in peroxisomes in which electrons removed from various metabolites reduce O_2_ to H_2_O_2_, which is further reduced to H_2_O. The high peroxisomal consumption of O_2_, the demonstration of the production of H_2_O_2_,  O_2_^•−^, ^•^OH, and recently of ^•^NO [6–9], and the discovery of several ROS metabolizing enzymes in peroxisomes has supported the notion that these ubiquitous organelles play a key role in both the production and scavenging of ROS in the cell [1].

Together with oxygen metabolism, peroxisomes fulfill multiple tasks [10]. The functional relevance of these organelles is dramatically highlighted in the nervous system by peroxisomal disorders. Genetic diseases classified as peroxisome biogenesis disorders and single peroxisomal enzyme deficiencies imply severe demyelination, axonal degeneration, and neuroinflammation that result in a variety of neurological abnormalities [11–15]. On the other hand, peroxisomes have recently been involved in cell aging [16] and in the development and progression of specific degenerative diseases [14, 17–22].

Since a common feature of several neurodegenerative diseases is inflammation [23], several studies have pointed to the potential use of agonists of the Peroxisome Proliferator Activated Receptor-*γ* (PPAR-*γ*). Increasing evidence demonstrates the neuroprotective effects of PPAR-*γ* agonists in a variety of preclinical models of neurological disorders such as Alzheimer's disease [24–26], Parkinson's disease [27], amyotrophic lateral sclerosis [28], Huntington's disease [29], and ischemic damage [30]. Nevertheless, evidence of PPAR-*γ* impairment in the physiopathology of neurodegenerative diseases is lacking, as well as the effects induced by its hypofunctionality in the nervous system. The theoretical basis of a PPAR-*γ* therapeutic approach in neurodegenerative disorders is generally founded on the anti-inflammatory effect. A clear relationship with peroxisome impairments is not well established. Although PPARs can transactivate genes pivotal for the functionality of these organelles [31, 32], the role of peroxisomes in PPAR-*γ* agonist efficacy, or in PPAR-*γ* hypofunction, remains unclear.

By focusing on astrocytes, glial cells strongly implicated in several degenerative diseases [33–35], we aimed to characterize the relevance of peroxisome functionality in PPAR-*γ*-dependent cell signaling. We have evaluated the damage evoked by PPAR-*γ* antagonists in a primary cell culture by analyzing characteristic peroxisome enzymes.

## 2. Material and Methods

### 2.1. Astrocyte Cultures

Primary cultures of astrocytes were obtained according to the method described by McCarthy and De Vellis [36]. Briefly, the cerebral cortex of newborn (P1–P3) Sprague-Dawley rats (Harlan, Udine, Italy) was dissociated in Hanks' balanced salt solution containing 0.5% trypsin/EDTA and 1% DNase (Sigma-Aldrich, Milan, Italy) for 30 min at 37°C. The suspension was mechanically homogenized and filtered. Cells were plated in high-glucose DMEM with 10% FBS. Confluent primary glial cultures were used to isolate astrocytes, removing microglia and oligodendrocytes by shaking. The purity of astrocyte cultures was determined immunocytochemically by staining for GFAP (Dako, Glostrup, Denmark). Cells were fixed in 4% paraformaldehyde, then incubated with the antibody (1 : 200), and visualized using Alexa Fluor-conjugated secondary antibody (Life Technologies, Monza, Italy). Nuclei were stained with 4,6-diamidino-2-phenylindole dihydrochloride. 90% of cells in astrocyte cultures were GFAP-positive. Experiments were performed 21 days after cell isolation. Formal approval to conduct the experiments described was obtained from the Animal Subjects Review Board of the University of Florence. The ethics policy of the University of Florence complies with the Guide for the Care and Use of Laboratory Animals of the US National Institutes of Health (NIH Publication number 85-23, revised 1996; University of Florence Assurance number A5278-01).

### 2.2. Catalase Activity

On day 21 of culture, astrocytes were plated in 12-well cell culture (2 · 10^5^/well; Corning, Tewksbury MA, USA) and experiments were performed after 48 h. Cells were treated with GW9662 (1–100 mM), G3335 (1–100 mM), and rosiglitazone (100 mM) for 2 or 5 days. All compounds were purchased from Sigma-Aldrich (Milan, Italy). After incubation, cells were washed once with PBS and scraped with PBS on ice. Cells were then collected, subjected to a freeze-thaw cycle, and centrifuged at 11,000 ×g for 10 min at 4°C. Catalase activity was measured in the supernatant by Amplex Red Catalase Assay Kit (Invitrogen, Monza, Italy) following the manufacturer's instructions. Protein concentration was quantified by bicinchoninic acid assay (Sigma-Aldrich, Milan, Italy). Catalase activity for each sample was normalized to protein concentration. Control conditions in the absence of treatment were set as 100%. Basal catalase activity was not different on days 0 (48 h after plating), 2, 4, 7, or 10 of culturing.

### 2.3. Hydrogen Peroxide Levels

On day 21 of culture, astrocytes were plated in 6-wells cell culture (5 · 10^5^/well; Corning, Tewksbury MA, USA) and experiments were performed 48 h after. After treatments with G3335 and rosiglitazone (2 and 5 days), cells were washed once with PBS and scraped with PBS on ice. Cells were then collected, subjected to a freeze-thaw cycle, and centrifuged at 11,000 ×g for 10 min at 4°C. Supernatants were treated with sorbitol to convert peroxide to a peroxyl radical, which oxidizes Fe^+2^ into Fe^+3^. Then the reaction between Fe^+3^ and an equal molar amount of xylenol orange in the presence of acid was allowed to create a purple product. The absorbance was read at 595 nm (OxiSelect Hydrogen Peroxide Assay Kit, Cell Biolabs, San Diego, CA, USA).

### 2.4. Western Blotting Analysis

On day 21 of culture, astrocytes were plated in 6-wells cell culture (5 · 10^5^/well; Corning, Tewksbury MA, USA) and experiments were performed 48 h after. Treatments with G3335 and rosiglitazone lasted 2 and 5 days. After incubation, cells were washed once with PBS and scraped on ice with lysis buffer containing 50 mM Tris-HCl pH 8.0, 150 mM NaCl, 1 mM EDTA, 0.5% Triton X-100, Complete Protease Inhibitor (Roche, Milan, Italy). Cells were then collected, subjected to a freeze-thaw cycle, and centrifuged at 11,000 ×g for 10 min at 4°C; the supernatant was conserved. Astrocyte protein extract was quantified by bicinchoninic acid assay and 40 *μ*g of each sample was resolved with 10% SDS-PAGE before electrophoretic transfer onto nitrocellulose membranes (Biorad, Milan, Italy). Membranes were blocked with 5% nonfat dry milk in PBS containing 0.1% Tween 20 (PBST) and then probed overnight at 4°C with primary antibody specific versus catalase (1 : 1000; 60 kDa; Novus Biological, Littleton, CO, USA), acyl-CoA oxidase 1 (ACOX1) (1 : 1000; 75 kDa; Santa Cruz Biotechnology, Santa Cruz, CA, USA), peroxisomal membrane protein of 70 kDa (PMP70) (1 : 1000; Abcam, Cambridge, MA, USA), glutathione reductase (1 : 1000; 65 kDa; Santa Cruz Biotechnology, Santa Cruz, CA, USA), NDUFS3, core subunit of Complex 1 NADH:ubiquinone oxidoreductase (1 : 1000; 30 kDa; Abcam, Cambridge, MA, USA), GAPDH (1 : 1000; 38 kDa; Cell Signaling, Boston, MA, USA), and *β*-Actin (1 : 1000; 42 kDa; Cell Signaling, Boston, MA, USA). Membranes were then incubated for 1 hour in PBST containing the appropriate horseradish peroxidase-conjugated secondary antibody (1 : 5000; Cell Signalling, Boston, MA, USA). ECL (Enhanced chemiluminescence Pierce, Rockford, IL, USA) was used to visualize the peroxidase-coated bands. Densitometric analysis was performed using the “ImageJ” analysis software (ImageJ, NIH, Bethesda, Maryland, USA) and results were normalized to GAPDH or *β*-Actin immunoreactivity as internal control. Values are reported as percentages in comparison to control which was arbitrarily fixed at 100%.

### 2.5. Statistical Analysis

Results are expressed as mean ± S.E.M. and analysis of variance (ANOVA) was performed. A Bonferroni significant difference procedure was used as post hoc comparison. All assessments were made by researchers blinded to cell treatments. *P* values of less than 0.05 were considered significant. Data were analyzed using the Origin 8.1 software (OriginLab, Northampton, MA, USA).

## 3. Results

The activity of the peroxisomal enzyme catalase was evaluated in astrocyte cell culture using a fluorometric assay.

**Figure 1:**
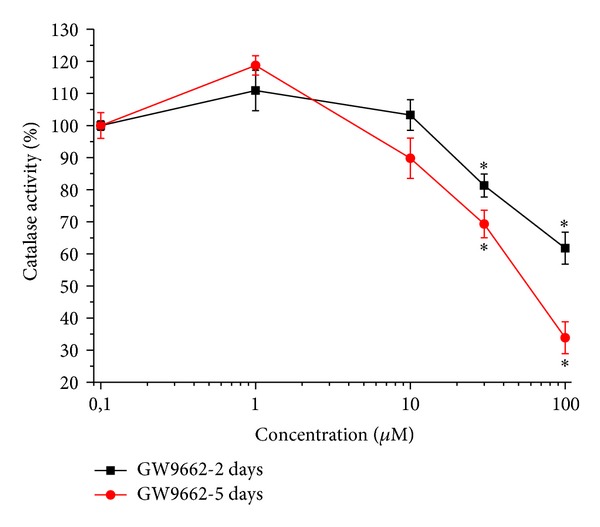
Catalase activity: effect of increasing concentrations of GW9662. Astrocytes (2 · 10^5^cells/well) were treated with the irreversible PPAR*γ* antagonist GW9662 (1–100 *μ*M) for 2 or 5 days. Values are expressed as the mean ± S.E.M. percent of control of three experiments. Control catalase activity was arbitrarily set as 100%. *P < 0.05 in comparison to control conditions in the absence of treatment.

The irreversible PPAR-*γ* antagonist GW9662 reduced catalase activity in a dose-dependent manner over time. As shown in Figure 1, a 2-day incubation with 30 *μ*M GW9662 decreased catalase activity to 81.4 ± 3.6% (control arbitrarily set at 100%), an effect comparable to that evoked by 100 *μ*M H_2_O_2_ after 2 h incubation (data not shown). The enzymatic activity decreased to 69.8 ± 2.8% after a 5-day incubation in the presence of 30 *μ*M GW9662 and to 32.7 ± 1.3% in the presence of 100 *μ*M GW9662 (Figure 1).

**Figure 2:**
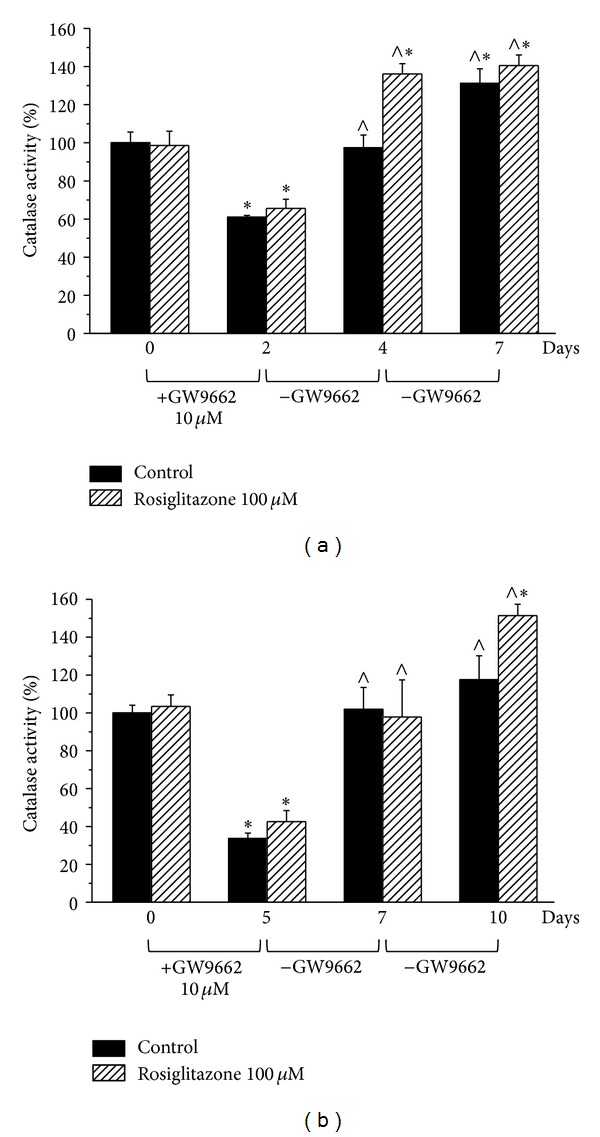
Restoration of catalase activity over time after treatment with GW9662. Astrocytes (2 · 10^5^ cells/well) were treated with the irreversible PPAR*γ* antagonist GW9662 (100 *μ*M) for (a) 2 days in the absence or presence of the PPAR*γ* agonist rosiglitazone (10 *μ*M). Cultures were continued for a further 2 or 5 days in the absence of GW9662 and in the absence or presence of rosiglitazone. Catalase activity was measured on days 0, 2, 4, and 7. (b) Cells were treated with GW9662 (100 *μ*M) for 5 days in the absence or presence of the PPAR*γ* agonist rosiglitazone (100 *μ*M). Cultures were allowed for further 2 or 5 days in the absence of GW9662 and in the absence or presence of Rosiglitazone. Catalase activity was measured on days 0, 5, 7, and 10. Values are expressed as the mean ± S.E.M. percent of control of three experiments. Control catalase activity (day 0) was arbitrarily set as 100%. *P < 0.05 in comparison to control conditions in the absence of treatment on day 0; ^∧^ *P* < 0.05 in comparison to control on days 2 (a) or 5 (b).

The activity impairment induced by 100 *μ*M GW9662 for 2 days (activity decreased to 61.0 ± 0.9%) was not prevented by the PPAR-*γ* agonist rosiglitazone (100 *μ*M) (Figure 2(a)). Allowing a further 2-day incubation in the absence of GW9662 (day 4), catalase activity was fully restored (97.4 ± 6.7%) and in the presence of rosiglitazone was stimulated up to 136.1 ± 5.4%. On day 7, which was 5 days after GW9662 washout, activity was about 140% both in the absence and presence of rosiglitazone.

The strong catalase activity decrease induced by a 5 day-incubation with 100 *μ*M GW9662 (Figure 2(b)) was restored by a 2-day washout (day 7). The stimulatory effect of rosiglitazone was significant on day 10 (151.4 ± 6.0%; Figure 2(b)).

**Figure 3:**
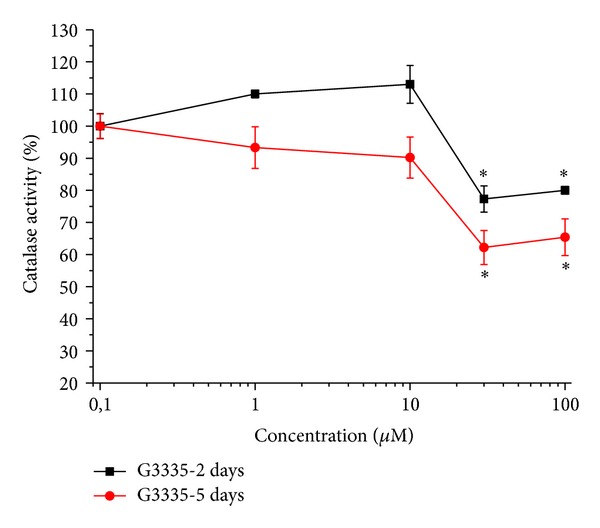
Catalase activity: effect of increasing concentrations of G3335. Astrocytes (2 · 10^5^cells/well) were treated with the reversible PPAR*γ* antagonist G3335 (1–100 *μ*M) for 2 or 5 days. Values are expressed as the mean ± S.E.M. percent of control of three experiments. Control catalase activity was arbitrarily set as 100%. *P < 0.05 in comparison to control conditions in the absence of treatment.

**Figure 4:**
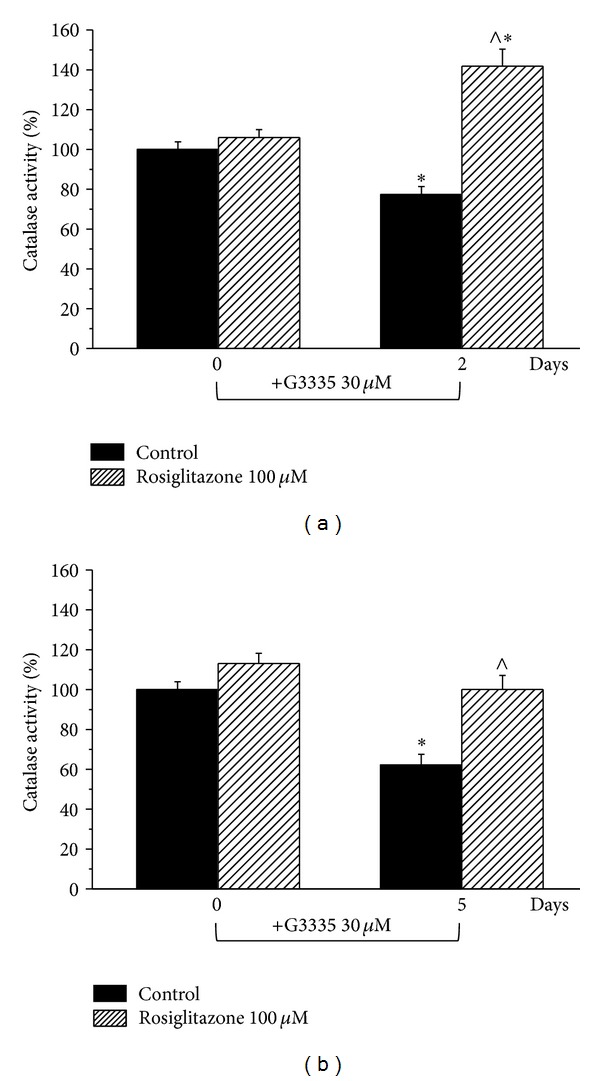
Catalase activity: effect of rosiglitazone on G3335-induced enzymatic impairment. Astrocytes (2 · 10^5^cells/well) were treated with the reversible PPAR*γ* antagonist G3335 (30 *μ*M) for (a) 2 days or (b) 5 days, in the absence or presence of the PPAR*γ* agonist rosiglitazone (100 *μ*M). Catalase activity was measured on days 0, 2, and 5. Values are expressed as the mean ± S.E.M. percent of control of three experiments. Control catalase activity (day 0) was arbitrarily set as 100%. *P < 0.05 in comparison to control conditions in the absence of treatment on day 0; ^∧^ *P* < 0.05 in comparison to control on days 2 (a) or 5 (b).

**Figure 5:**
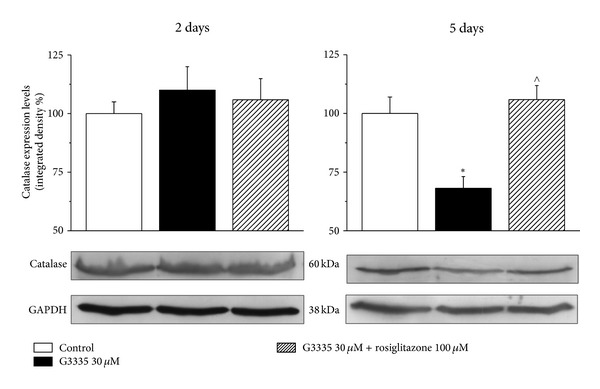
Catalase expression: effect of G3335. Astrocytes (5 · 10^5^cells/well) were treated with the reversible PPAR*γ* antagonist G3335 (30 *μ*M) for 2 days or 5 days, in the absence or presence of the PPAR*γ* agonist rosiglitazone (100 *μ*M). Values are expressed as the mean ± S.E.M. percent of control of three experiments. Control condition was arbitrarily set as 100%. *P < 0.05 in comparison to control conditions in the absence of treatment; ^∧^ *P* < 0.05 in comparison to G3335 treatment on day 5.

**Figure 6:**
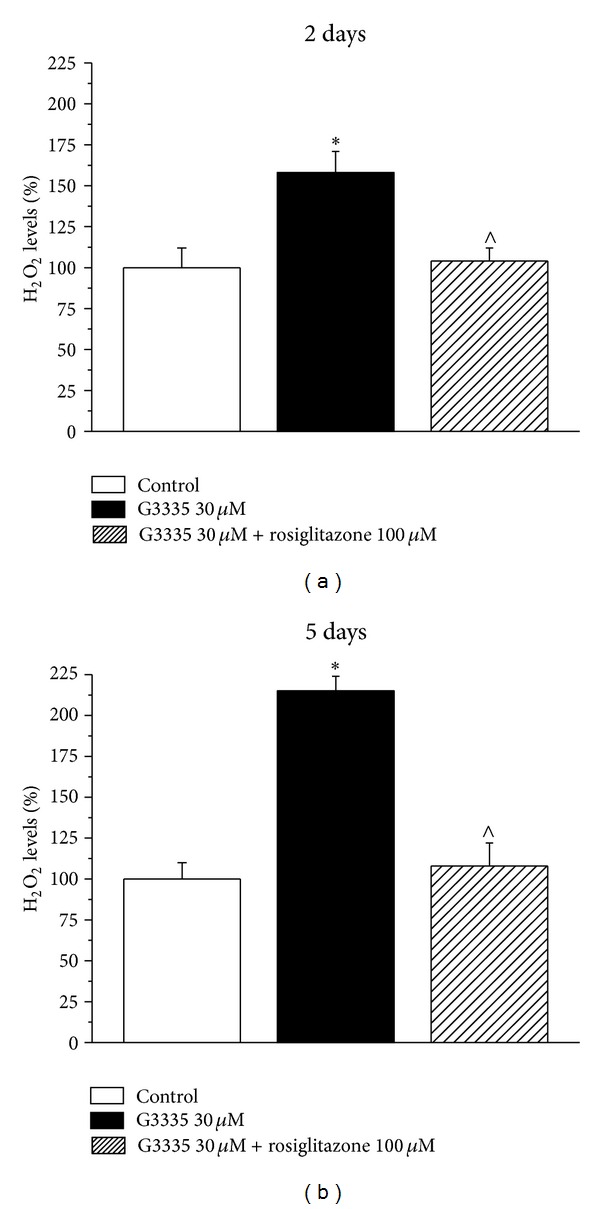
Hydrogen peroxide levels: effect of G3335. Astrocytes (5 · 10^5^cells/well) were treated with the reversible PPAR*γ* antagonist G3335 (30 *μ*M) for 2 days or 5 days, in the absence or presence of the PPAR*γ* agonist rosiglitazone (100 *μ*M). Values are expressed as the mean ± S.E.M. percent of control of three experiments. Control condition was arbitrarily set as 100%. *P < 0.05 in comparison to control conditions in the absence of treatment; ^∧^ *P* < 0.05 in comparison to G3335 treatment on days 2 or 5.

As shown in Figure 3, the reversible PPAR-*γ* antagonist G3335 (30 *μ*M) induced catalase impairment (after a 2-day incubation 77.3 ± 4.1%; after 5 days 62.2 ± 5.3%) comparable to that evoked by GW9662 but this effect seems to be maximal since a higher concentration (100 *μ*M) did not increase the damage (Figure 4). G3335-dependent catalase damage was prevented in the presence of 100 *μ*M rosiglitazone both at 2 and 5 days (Figures 4(a) and 4(b)). Rosiglitazone was also able to improve catalase activity over 100% after a 2-day incubation (Figure 4(a)). The expression level of catalase was unaltered by 30 *μ*M G3335 after a 2-day incubation. On the contrary, a 30% decrease induced by 5 days' incubation of G3335 was fully prevented by 100 *μ*M rosiglitazone (Figure 5). Catalase impairment was associated with a time-dependent increase of hydrogen peroxide levels (about 50 and 100% after 2- and 5-day incubation, resp., Figure 6). In the presence of rosiglitazone H_2_O_2_ levels were normalized.

**Figure 7:**
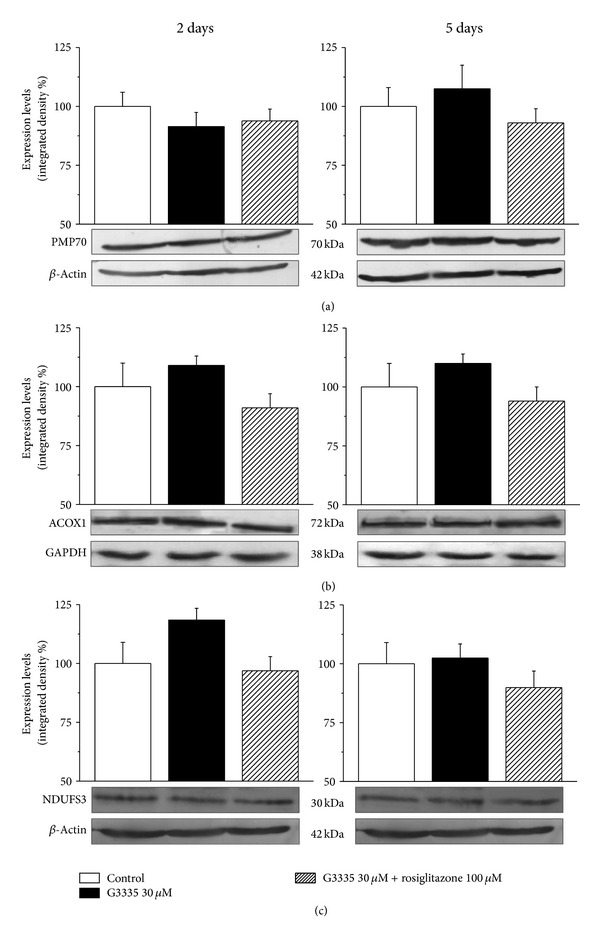
(a) PMP70, (b) ACOX1, and (c) NDUFS3 expression: effect of G3335. Astrocytes (5 · 10^5^cells/well) were treated with the reversible PPAR*γ* antagonist G3335 (30 *μ*M) for 2 days or 5 days, in the absence or presence of the PPAR*γ* agonist rosiglitazone (100 *μ*M). Values are expressed as the mean ± S.E.M. percent of control of three experiments. Control condition was arbitrarily set as 100%.

G3335 did not alter PMP70 expression levels at both times evaluated (2 and 5 days, Figure 7(a)). G3335 did not alter ACOX1 expression levels at both times evaluated (2 and 5 days, Figure 7(b)). Expressions of the antioxidant enzyme glutathione reductase and Complex 1 NADH dehydrogenase were also measured to evaluate the protective response of astrocytes to the presence of G3335. The NDUFS3 subunit of the mitochondrial enzyme Complex 1 expression was not modified by G3335 (Figure 7(c)).

**Figure 8:**
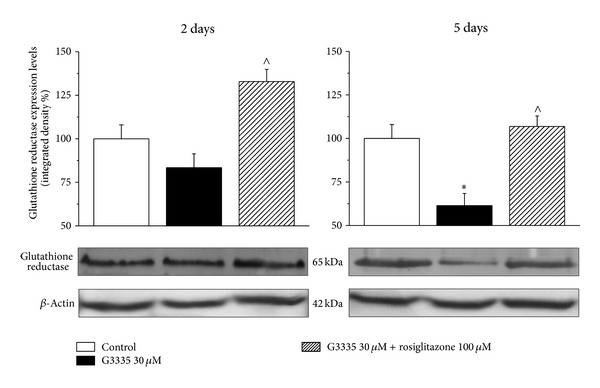
Glutathione reductase expression: effect of G3335. Astrocytes (5 · 10^5^cells/well) were treated with the reversible PPAR*γ* antagonist G3335 (30 *μ*M) for 2 days or 5 days, in the absence or presence of the PPAR*γ* agonist rosiglitazone (100 *μ*M). Values are expressed as the mean ± S.E.M. percent of control of three experiments. Control condition was arbitrarily set as 100%. *P < 0.05 in comparison to control condition in the absence of treatment; ^∧^ *P* < 0.05 in comparison to G3335 treatment on days 2 or 5.

Glutathione reductase expression was progressively reduced by 30 *μ*M G3335 over time. After 5 days' incubation, protein levels decreased by about 40% (Figure 8), whereas 100 *μ*M rosiglitazone prevented this effect (Figure 8).

## 4. Discussion

PPARs are members of the nuclear receptor superfamily, actively involved in immunoregulation through their ability to regulate membrane lipid composition, cell proliferation, sensitivity to apoptosis, energy homeostasis, and various inflammatory transcription factors, mainly through their transrepression capabilities [23]. Although all three subtypes of PPARs (*α*, *β*/*δ*, and *γ* [37]) have been implicated in brain damage, PPAR-*γ* is the most extensively studied [23, 38–40]. PPAR-*γ* agonists may ameliorate AD-related pathology and improved learning and memory in animal models and memory and cognition in AD patients [24–26]. Activation of PPAR-*γ* by pioglitazone induces behavioral recovery associated with preservation of nigrostriatal dopaminergic markers and reduction of CD68-positive cells in Parkinsonian monkeys [27]. PPAR-*γ* agonists have beneficial effects in an experimental model of Huntington's disease by interfering with the NF-*κ*B signaling pathway [29]. Heneka et al. [30] showed that rosiglitazone delays neuronal damage by interfering with glial activations and increases anti-inflammatory cytokines in response to ischemic damage. On the other hand, there is scanty knowledge about the pathophysiological effects induced by PPAR-*γ* dysfunctions. In the present results a relationship between PPAR-*γ* inhibition in astrocytes and peroxisomal function impairment is shown. The irreversible PPAR-*γ* antagonist GW9662 concentration-dependently decreases catalase activity up to 30%. As expected, GW9662-dependent impairment is not prevented by the PPAR-*γ* agonist rosiglitazone. On the contrary, catalase functionality recovers in a few days in cell culture in the absence of GW9662, suggesting the plasticity of peroxisome in adverse conditions. Rosiglitazone stimulates the physiological restoration of the enzymatic damage leading to the hypothesis that PPAR-*γ* agonists may positively intervene in rescue signaling. The reversible antagonist G3335 reduces progressively catalase activity to 60% reaching a plateau for concentrations higher than 30 *μ*M. Two days of incubation are needed for enzyme hypofunctionality, and decreased expression follows 3 days later. Both activity and expression reduction of catalase are prevented by rosiglitazone.

Catalase is the most important antioxidant defense enzyme in mammalian peroxisomes. In rodent liver peroxisomes, rough estimates indicate that each molecule of H_2_O_2_-producing oxidase possesses at least one molecule of catalase as a functional counterpart [41]. Considering that mammalian peroxisomes are densely populated by enzymes that form ROS (most of them are FAD- or FMN-dependent oxidases generating H_2_O_2_; [42]) it is not surprising that peroxisomes are well equipped with antioxidant defense systems composed mainly of enzymes involved in the decomposition of H_2_O_2_ [43]. Catalase impairment has been observed in neurodegenerative conditions [44] as well as in complex neurodevelopmental disorders such as autism spectrum, whose neurobiology is proposed to be associated with oxidative stress [45]. On the other hand, oxidative stress may result from an increase in ROS generation as well as from an impairment of catabolic phenomena. Alterations in consumer enzymes may vary the net rate between production and consumption and induce a release of ROS from the organelles to the cell [46]. H_2_O_2_, unlike O_2_^•−^, is able to cross membranes and is free to leave the organelle and to induce cell damage [43]. On the contrary, catalase stimulation is protective against nervous injuries [47]. In the present results, a progressive increase of H_2_O_2_ parallels with catalase hypofunction.

Conversely, G3335-induced PPAR-*γ* block does not alter the expression of another major peroxisome protein, PMP70, a membrane protein possessing multiple peroxisome-targeting signals [48]. PMP70 is a half-type ABC-transporter [49] involved in the transport of long and branched chain acyl-CoA [50]. These data suggest lack of a relationship between PPAR-*γ* and PMP70 in astrocytes.

*β*-oxidation of a number of carboxylates that cannot be handled by mitochondria is one of the most important metabolic reactions occurring in peroxisomes [51, 52] and this process also contributes to the formation of H_2_O_2_ [53, 54]. ACOX1 catalyzes the first and rate-limiting step of straight-chain fatty acid *β*-oxidation [55, 56]. ACOX1 is considered to be a PPAR-*α* target gene predictive of peroxisome proliferation [57, 58] but, given that PPAR subtypes recognize and activate gene expression through a common DNA binding site [59], ACOX1 could be regulated also by PPAR-*γ*. In the present results, G3335 does not modify the full length ACOX1 protein expression levels in astrocytes suggesting a specific regulation of PPAR-*γ* target genes.

To fulfill their functions, peroxisomes physically and functionally interact with other cell organelles, including mitochondria, the endoplasmic reticulum, and lipid droplets [1, 60]. It is well established that peroxisomes and mitochondria are metabolically linked in mammals [61]. Disturbance in peroxisomal metabolism triggers signaling/communication events that ultimately result in increased mitochondrial stress [62, 63]. To evaluate the effect of PPAR-*γ*  inhibition on a characteristic enzyme of the detoxificant machinery of mitochondria we evaluated the expression levels of NDUFS3, a core subunit of Complex 1, the first and largest of the four multiprotein complexes that constitute the mitochondrial respiratory chain involved in oxidative phosphorylation [64]. In particular, NDUFS3 primarily initiates the *in vivo* assembly of Complex 1 in the mitochondrial matrix [65]. Our results show that G3335 does not alter NDUFS3 expression in astrocytes, suggesting that these conditions are specific to peroxisomal damage. However, peroxisome impairment is enough to decrease glutathione reductase expression levels in a rosiglitazone-prevented manner. Glutathione reductase generates reduced glutathione, the main protector of the cell [66] and low levels of this enzyme may have implications for oxidative stress. Glutathione reductase has been described to be reduced in neurodegenerative diseases like PD [67], AD [44], adrenoleukodystrophy [66], and amyotrophic lateral sclerosis [68]. Astrocytes exert neuroprotective effects by providing neurons with substrates for antioxidants such as glutathione [69]. Astrocytes contain high levels of antioxidant molecules such as vitamins E and C and the antioxidant enzymes Mn- and Cu, Zn-superoxide dismutases (Mn- and Cu, Zn-SOD), catalase, and glutathione peroxidase, which play a major neuroprotective role against the deleterious effects of ROS [70, 71]. Although astrocytes are generally less susceptible to oxidative injury than neurons, there is strong evidence that oxidative stress also alters astrocyte functions [40, 72]. In particular, glial cells are extremely vulnerable to H_2_O_2_ and astrocytic apoptosis is observed in brain injuries caused by trauma and ischemia [73, 74] and in models of neuropathies [75]. Protection of astrocytes from oxidative attack appears essential to maintain cerebral antioxidant competence and to prevent neuronal damage as well as to facilitate neuronal recovery [76]. It has been shown that peroxisomes provide glial cells with neuroprotective and anti-inflammatory functions [77] and loss or impairment of peroxisomal function results in characteristic patterns of central nervous system lesions [11, 12]. This is best illustrated by pathomorphological examinations of the brain of patients (and mice) in which one or more peroxisomal functions are lost [12, 77–81].

## 5. Conclusion

In this report we highlight that the PPAR-*γ* block in astrocytes is strictly related to reduced catalase functionality and expression with a general decrease in antioxidant defenses of the cell. The relevance of the damage induced by PPAR-*γ* impairment suggests that hypofunctionality of this receptor in glial cells could be present in neurodegenerative diseases and participate in pathological mechanisms through peroxisomal damage. The present series of experiments could offer a useful model for the study of PPAR-*γ* agonists or, in general, compounds able to restore peroxisome functionality.
